# COVID-19 mRNA Vaccination: Age and Immune Status and Its Association with Axillary Lymph Node PET/CT Uptake

**DOI:** 10.2967/jnumed.121.262194

**Published:** 2022-01

**Authors:** Michal Eifer, Noam Tau, Yousef Alhoubani, Nayroz Kanana, Liran Domachevsky, Jala Shams, Nir Keret, Malka Gorfine, Yael Eshet

**Affiliations:** 1 Department of Diagnostic Imaging, Chaim Sheba Medical Center, Ramat Gan, Israel;; 2 Sackler Faculty of Medicine, Tel-Aviv University, Tel Aviv, Israel; and; 3 Department of Statistics and Operations Research, Tel-Aviv University, Tel Aviv, Israel

**Keywords:** COVID-19, mRNA vaccine, PET/CT, axillary lymphadenopathy, immunogenicity

## Abstract

With hundreds of millions of coronavirus disease 2019 (COVID-19) messenger RNA (mRNA)–based vaccine doses planned to be delivered worldwide in the upcoming months, it is important to recognize PET/CT findings in recently vaccinated immunocompetent or immunocompromised patients. We aimed to assess PET/CT uptake in the deltoid muscle and axillary lymph nodes of patients who received a COVID-19 mRNA-based vaccine and to evaluate its association with patient age and immune status. **Methods:** All consecutive adults who underwent PET/CT scans with any radiotracer at our center during the first month of a national COVID-19 vaccination rollout (between December 23, 2020, and January 27, 2021) and had received the vaccination were included. Data on clinical status, laterality, and time from vaccination were prospectively collected, retrospectively analyzed, and correlated with deltoid muscle and axillary lymph node uptake. **Results:** Of 426 eligible subjects (median age, 67 ± 12 y; 49% female), 377 (88%) underwent PET/CT with ^18^F-FDG, and positive axillary lymph node uptake was seen in 45% of them. Multivariate logistic regression analysis revealed a strong inverse association between positive ^18^F-FDG uptake in ipsilateral lymph nodes and patient age (odds ratio [OR], 0.57; 95% CI, 0.45–0.72; *P* < 0.001), immunosuppressive treatment (OR, 0.37; 95% CI, 0.20–0.64; *P* = 0.003), and presence of hematologic disease (OR, 0.44; 95% CI, 0.24–0.8; *P* = 0.021). No such association was found for deltoid muscle uptake. The number of days from the last vaccination and the number of vaccine doses were also significantly associated with increased odds of positive lymph node uptake. **Conclusion:** After mRNA-based COVID-19 vaccination, a high proportion of patients showed ipsilateral lymph node axillary uptake, which was more common in immunocompetent patients. This information will help with the recognition of PET/CT pitfalls and may hint about the patient’s immune response to the vaccine.

## Visual Abstract

**Figure:**
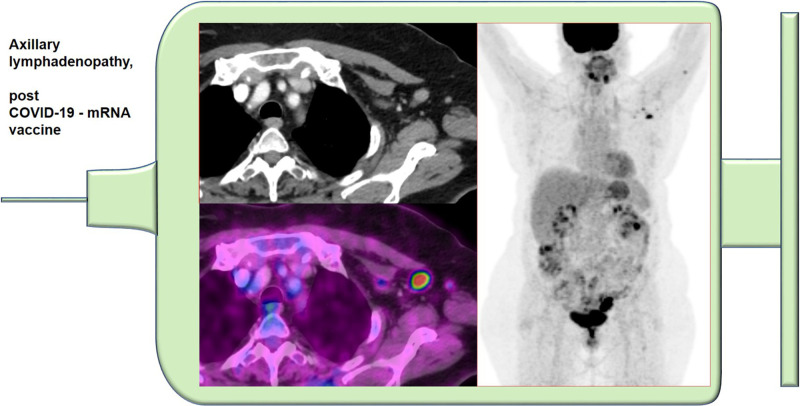

The emergence of pneumonia cases caused by the novel severe acute respiratory syndrome coronavirus 2 in December 2019 (*1*) and the subsequent coronavirus disease 2019 (COVID-19) pandemic have led to initiatives for developing an effective vaccine from as early as January 2020. To date, more than 230 vaccine candidates have been developed (*2*), many of them using innovative vaccine development technologies, most of which had never been used commercially in humans before (*3*).

The first vaccines to be approved by the U.S. Food and Drug Administration, in December 2020, were based on a messenger RNA (mRNA) sequence encoding segments of the spike protein of the severe acute respiratory syndrome coronavirus 2 and encapsulated in lipid nanoparticles (*4*,*5*).

To date, there are no published data regarding the efficacy of mRNA vaccines in immunocompromised populations, and both the U.S. Centers for Disease Control and Prevention and the U.S. Food and Drug Administration state that immunocompromised persons receiving mRNA vaccines may have a diminished immune response (*4*–*6*).

As the global COVID-19 vaccination endeavor is in its early days, there are no published data on the prevalence of post–mRNA vaccination PET/CT findings, especially in oncologic patients, either immunocompetent or immunocompromised. The most common findings described in patients who underwent PET/CT after receiving vaccines against influenza or papillomavirus were ipsilateral lymphadenopathy with varying degrees of uptake (*7*–*15*).

In this study, we aimed to describe the PET/CT findings in patients after mRNA-based COVID-19 vaccination and to identify the patient characteristics associated with PET/CT uptake.

## MATERIALS AND METHODS

### Study Design and Setting

We conducted a retrospective analysis of prospectively collected data. The study was approved by the institutional ethics committee. The need for patient informed consent was waived.

### Patients

All consecutive adults (>18 y old) who had been referred for a PET/CT scan (with any radiotracer) for any indication between December 23, 2020 (the initiation date of a national COVID-19 vaccination rollout in the general population), and January 27, 2021, and had received the vaccination were included in the study. Patients were excluded if they had incomplete medical records or a known malignancy involving axillary lymph nodes.

### Data Collection

Before entering the PET/CT unit, all patients were asked to complete a standard clinical intake form, which included age, sex, indication for PET/CT, clinical and oncologic status, current oncologic treatment, recent surgical procedures (including location of the procedure), date of COVID-19 vaccination, and arm in which the vaccine was administered for the first and second (if applicable) doses.

Immunosuppressive status was assigned to patients with a known hematologic disease (any type of lymphoma, leukemia, or multiple myeloma) or receiving any of the following treatments considered to result in immunosuppression: current corticosteroid therapy, chemotherapy within the last 3 mo, treatment with rituximab or daratumumab in the last 6 mo, or bone marrow transplantation within the last 6 mo (*16*).

### PET/CT Acquisition and Analysis

All PET/CT scans were performed according to our institute’s clinical scanning protocols. A diagnostic CT examination was performed on a 64-detector-row helical CT scanner (Vereos; Philips). The field of view and pixel size for PET images reconstructed for fusion were 57.6 cm and 4 mm, respectively, with a matrix size of 144 × 144. The technical parameters used for CT imaging were a pitch of 0.83, a gantry rotation speed of 0.5 s/rotation, 120 kVp, a modulated tube current of 40–300 mA, and specific breath-holding instructions. After fasting for 2–6 h, the patients received an intravenous injection of tracer: 5.18 MBq/kg for ^18^F-FDG or ^18^F-fluorodopa (^18^F-DOPA), 3.7 MBq/kg for ^18^F-prostate-specific membrane antigen (^18^F-PSMA), and 185–296 MBq for ^68^Ga-PSMA or ^68^Ga-DOTATATE. About 60 min after tracer administration, CT images were obtained from the vertex to the mid thigh or of the whole body. An emission PET scan followed in 3-dimensional acquisition mode for the same longitudinal coverage, 1.5 min per bed position. CT images were fused with the PET data and used to generate a map for attenuation correction, eventually generating reconstructed images for review on a computer workstation.

The images were analyzed using Vue PACS (version 12.1.5.1; Carestream). All PET/CT images were read in consensus by a physician with dual board certification in radiology and nuclear medicine and 6 y of PET/CT reading experience, 2 board-certified radiologists in nuclear medicine residency with 3 y of PET/CT experience, and a nuclear medicine resident with 1 y of PET/CT experience.

Deltoid muscle and axillary lymph node SUV_max_, normalized for body weight, was measured by placing a region of interest at the injection site and at the draining axillary lymph node with the highest uptake (i.e., the ipsilateral side), as well as on the contralateral deltoid muscle and axillary lymph node (i.e., the contralateral side), which were used as a reference.

Because SUV_max_ depends on the tracer used and on variable technical aspects, uptake in the deltoid muscle and axillary lymph node was defined as positive if the ipsilateral-to-contralateral SUV_max_ ratio was at least 1.5, a method previously used by Thomassen et al. (*9*).

### Statistical Analysis

Logistic regression models were fit with a binary dependent variable (positive deltoid muscle or axillary lymph node uptake [yes/no]) and with the following independent variables: scaled age, sex, immunosuppressive treatment (yes/no), hematologic disease (yes/no), scaled days since last vaccine dose, whether a second vaccine dose was administered (yes/no), and the interaction between the last 2 independent variables. The test of Hosmer and Lemeshow (*17*) with 8 groups (representing the number of covariates plus 1) was used for assessing the goodness of fit of each model. The Benjamini and Hochberg false-discovery rate method (*18*) was used for multiple-testing adjustment, jointly, for the 2 models, at the level of 0.05. All statistical analyses were conducted in the R environment (https://www.r-project.org/). Because of small number of non–^18^F-FDG scans, only ^18^F-FDG scans were included in the statistical analysis.

## RESULTS

**FIGURE 1.**
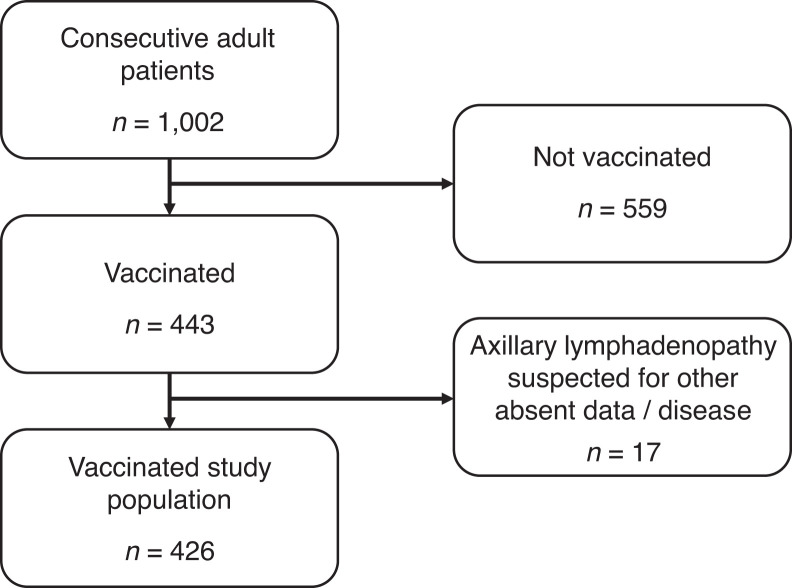
Patient flowchart.

Of 1,002 consecutive adults scanned during the study, 44% (443) received at least 1 vaccine dose before the scan. Of those vaccinated, 23% (103/443) received the second vaccine dose. After excluding patients with incomplete medical records or with a known malignancy involving the axillary lymph nodes, the final vaccinated study cohort comprised 426 patients with a mean age of 67 y (SD, 12), each with a single PET/CT scan (Fig. 1). Patient demographics are shown in Table 1. Most patients (377/426, 88%) underwent ^18^F-FDG scans. ^18^F-PSMA and ^68^Ga-PSMA scans were performed on 37 patients (9%), ^68^Ga-DOTATATE scans on 11 patients (2.5%), and an ^18^F-DOPA scan on 1 patient (0.5%). Among those who underwent ^18^F-FDG PET/CT, 20% (76/377) received a second vaccine dose, 62% (232/377) were immunocompetent, and 38% (145/377) were immunocompromised. Among the immunocompromised patients, 52% (75/145) had a hematologic malignancy and 56% (82/145) received immunosuppressive therapy. There was an overlap between these 2 groups, as 12 hematologic patients also received immunosuppressive treatment.

**TABLE 1.** Demographic Characteristics of Vaccinated Study Population

Variable	Data
Mean age ± SD (y)	67 ± 12 (range, 20–95)
Female	207 (49%)
PET/CT scan indication	
Solid tumor	357 (83%)
Hematologic malignancy	66 (16%)
Inflammation or infection	4 (1%)
Treatment*	
No current treatment	235 (55%)
Targeted therapy^†^	74 (17%)
Chemotherapy	71 (16%)
Immunotherapy	48 (11%)
Steroids	27 (6%)
Other immunosuppressive treatment^‡^	4 (1%)

*Some patients received more than 1 treatment.

^†^Tyrosine kinase inhibitors, hormonal therapy, or proteasome inhibitors.

^‡^Rituximab, daratumumab, or bone marrow transplantation.

Data are number followed by percentage in parentheses, except for age Total *n* = 426.

The time between the first vaccine dose and the PET/CT scan ranged between 1 and 34 d (median, 13 d). The median time between the last vaccination dose and the PET/CT scan was 11 d (SD, ± 6.4) for patients who received only 1 vaccine dose and 4 d (SD, ± 3.8) for patients who received a second vaccine dose. Although the overall time frame between vaccination and imaging was large (1–34 d), the distribution was similar between the immunocompromised and immunocompetent groups (interquartile ranges, 6–21 and 6–20, respectively).

**FIGURE 2.**
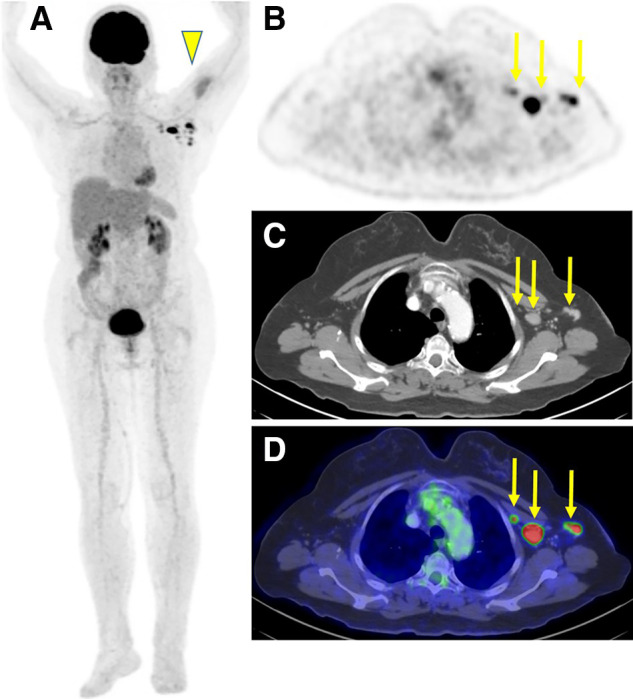
^18^F-FDG PET/CT after COVID-19 vaccination in 66-y-old woman with suspected colon cancer and elevated carcinoembryonic antigen, with no current immunosuppressive treatment: maximal-intensity projection (A), axial multiplanar reformation (B), contrast-enhanced CT (C), and PET/CT (D) 25 d after first vaccine dose and 2 d after second vaccine dose, both in left arm. Increased uptake was observed in left deltoid muscle (arrowhead), corresponding to injection site, and in ipsilateral enlarged axillary lymph nodes (arrows). Otherwise, there were no hypermetabolic findings suggestive of malignancy.

**FIGURE 3.**
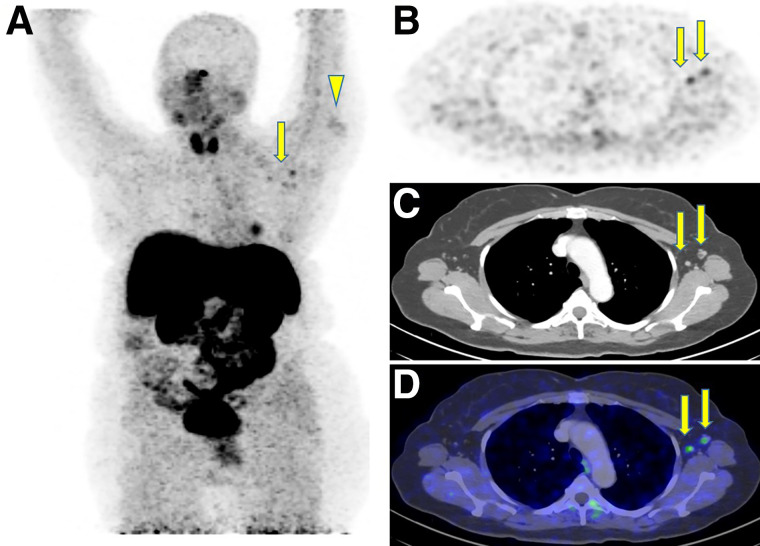
^68^Ga-DOTATATE PET/CT after COVID-19 vaccination in 68-y-old woman with newly diagnosed typical carcinoid (G2), not currently receiving immunosuppressive treatment: maximal-intensity projection (A), axial multiplanar reformation (B), contrast-enhanced CT (C), and PET/CT (D) 24 d after first vaccine and 3 d after second vaccine, both on left side. Increased uptake was observed in left deltoid muscle (arrowhead), corresponding to injection site, and in left axillary lymph nodes of normal size (arrows).

Although positive ^18^F-FDG and ^68^Ga-DOTATATE axillary lymph node uptake was noted in about half the patients, ^18^F-PSMA, ^68^Ga-PSMA, and ^18^F-DOPA uptake was far less common (Table 2; Figs. 2 and 3). The mean ^18^F-FDG SUV_max_ in patients with positive uptake was 2 ± 0.8 (range, 0.6–4.6) and 2.7 ± 1.6 (range, 0.6–12.4) in the deltoid muscle and axillary lymph nodes, respectively.

**TABLE 2.** Prevalence of Increased Uptake in Ipsilateral Deltoid Muscle or in Ipsilateral Axillary Lymph Nodes with Different PET/CT Tracers

Tracer	Deltoid muscle uptake	Axillary lymph node uptake	Both deltoid and axillary uptake
^18^F-FDG	26% (98/377)	45% (170/377)	16% (60/377)
^68^Ga-DOTATATE	9% (1/11)	55% (6/11)	9% (1/11)
^68^Ga- or ^18^F-PSMA	0% (0/37)	0.3% (1/37)	0% (0/37)
^18^F-DOPA	0% (0/1)	100% (1/1)	0% (0/1)

A little over half the immunocompetent patients (122/232, 53%) and a third of the immunocompromised patients (48/145, 33%) showed ^18^F-FDG axillary lymph node uptake. More specifically, ^18^F-FDG axillary lymph node uptake was observed in 30% (25/82) of patients treated with immunosuppressive treatment and 32% (24/75) of patients with a hematologic disease.

The multivariate logistic regression analysis of SUV uptake in the deltoid muscle and the lymph nodes after COVID-19 vaccination is summarized in Table 3. There was a strong inverse association between positive ^18^F-FDG uptake in ipsilateral lymph nodes and patient age (odds ratio [OR], 0.57; 95% CI, 0.45–0.72; *P* < 0.001), immunosuppressive treatment (OR, 0.37; 95% CI, 0.20–0.64; *P* = 0.003), and presence of hematologic disease (OR, 0.44; 95% CI, 0.24–0.8; *P* = 0.021). In addition, the number of days from the last vaccine dose and the number of vaccine doses were significantly associated with increased odds of positive lymph node uptake (OR, 1.53; 95% CI, 1.18–1.99[*P* = 0.005], and OR, 7.53; 95% CI, 2.91–23.50 [*P* = 0.001], respectively). No association was found between positive deltoid muscle uptake and patient age (OR, 0.86; 95% CI, 0.66–1.12; *P* = 0.32), immunosuppressive treatment (OR, 0.63; 95% CI, 0.31–1.23; *P* = 0.277), or hematologic disease (OR, 0.72; 95% CI, 0.34–1.42; *P* = 0.411). The number of vaccine doses was also associated with increased odds for positive deltoid muscle uptake (OR, 2.85; 95%CI, 1.13–6.70; *P* = 0.040), whereas the interaction between the number of vaccine doses and the number of days from the last vaccination was associated with decreased odds of positive deltoid muscle uptake (OR, 0.28; 95%CI, 0.09–0.75; *P* = 0.036).

**TABLE 3.** Multivariate Logistic Regression Analysis of Uptake in Deltoid Muscle and Lymph Nodes After COVID-19 mRNA Vaccination

Independent variable	OR	95% CI	*P*	Adjusted P*
Deltoid muscle^†^				
Scaled age	0.86	0.66–1.12	0.251	0.320
Sex (male)	1.09	0.64–1.85	0.744	0.744
Immunosuppressive treatment (yes)	0.63	0.31–1.23	0.192	0.277
Hematologic disease (yes)	0.72	0.34–1.42	0.352	0.411
Scaled number of days from last vaccination	0.74	0.53–1.01	0.066	0.116
Second vaccination (yes)	2.85	1.13–6.70	0.020	0.040*
Scaled number of days from last vaccination: second vaccination	0.28	0.09–0.75	0.015	0.036*
Constant	0.23			
Lymph nodes^†^				
Scaled age	0.57	0.45–0.72	0.000	0.000*
Sex (male)	0.74	0.47–1.17	0.198	0.277
Immunosuppressive treatment (yes)	0.37	0.20–0.64	0.001	0.003*
Hematologic disease (yes)	0.44	0.24–0.80	0.008	0.021*
Scaled number of days from last vaccination	1.53	1.18–1.99	0.001	0.005*
Second vaccination (yes)	7.53	2.91–23.50	0.000	0.001*
Scaled number of days from last vaccination: second vaccination	1.39	0.50–4.43	0.552	0.594
Constant	0.93			

*P < 0.05 (false-discovery rate–adjusted for multiple testing).

^†^Hosmer–Lemeshow tests showed no indication of poor fit (*P* = 0.919 for deltoid muscle model, *P* = 0.674 for lymph node model).

**FIGURE 4.**
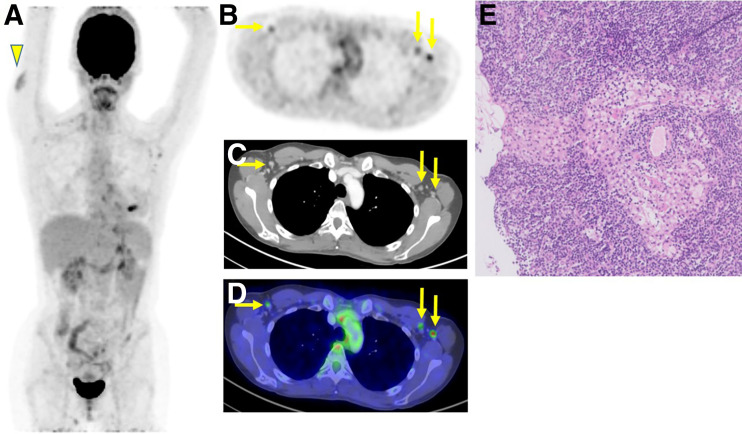
Pathology of reactive, ^18^F-FDG–avid, axillary lymph node after COVID-19 vaccination in 41-y-old woman with newly diagnosed left-sided estrogen receptor–positive, progesterone receptor–positive, human epidermal growth factor receptor 2–positive breast cancer: maximal-intensity projection showing marked increase in ^18^F-FDG uptake in right deltoid muscle (arrowhead), corresponding to recent second vaccine dose site, and in several left and right axillary lymph nodes (A); axial multiplanar reformation (B); contrast-enhanced CT (C); and PET/CT showing marked bilateral increase in ^18^F-FDG uptake in axillary lymph nodes (arrows) (D). First vaccine dose was in left deltoid muscle before diagnosis of breast cancer and 22 d before scan. Second vaccine dose was in right deltoid muscle after diagnosis and 1 d before scan. Patient underwent ultrasound-guided core-needle biopsy to left suggestive axillary lymph node 1 d after PET/CT scan. (E) Hematoxylin- and eosin-stained images of cores of lymph node tissue showing prominently dilated and edematous sinuses that probably reflect reactive changes. Lymphoid tissue is unremarkable, and there is no evidence of malignancy.

A single patient presenting for staging of left-breast cancer after 2 doses of vaccine (first dose in left arm and second dose in right arm) had ^18^F-FDG–avid lymphadenopathy in the left axilla, but a further biopsy negated lymph node involvement (Fig. 4).

## DISCUSSION

With the global vaccination effort against COVID-19 under way, data on the safety and efficacy of the newly approved vaccines are accumulating (*19*). In the current study, 45% of patients demonstrated avid ipsilateral axillary lymphadenopathy on ^18^F-FDG PET/CT in the weeks after vaccination with the novel mRNA-based COVID-19 vaccine, a finding significantly less common in immunocompromised patients. During Pfizer’s phase 3 vaccine approval trial, only 0.3% of vaccinated participants were diagnosed with ipsilateral lymphadenopathy (*4*), suggesting that most lymph node uptake occurs in subclinical lymphadenopathy.

In our patient population, we encountered 426 patients who, over a short period of 5 wk, had received an mRNA vaccination for COVID-19 and undergone PET/CT (44% of all patients scanned). It soon became apparent that on many of those scans, the ipsilateral deltoid muscle and axillary lymph node showed avid uptake that was not related to the underlying disease (*20*).

Previous studies have explored the association between aging or immune status and vaccine-induced protection. Such studies showed impaired primary and secondary antibody responses to vaccination in the elderly (*21*), low vaccine-induced protection against influenza A (H1N1) in patients receiving anti-CD20 treatment with rituximab (*22*), and a weaker antibody response to influenza virus vaccine in patients receiving cancer chemotherapy (*23*). A previous study failed to show an association between ^18^F-FDG axillary lymph node uptake and chemotherapy in patients vaccinated against influenza virus (*8*). Our study demonstrated that in the weeks after injection of the novel mRNA-based vaccine, the ^18^F-FDG PET/CT images of 53% of immunocompetent patients showed avid ipsilateral axillary lymphadenopathy, a finding that was less common in immunocompromised patients (33%). Furthermore, we found a strong inverse association between axillary lymph node uptake, patient age, and immune status, as well as a strong association between the number of administered vaccine doses (the odds of positive lymph node uptake were higher in patients receiving 2 vaccine doses) and the time since the last vaccination (the longer the interval, the higher the odds of positive lymph node uptake). Conversely, no association was found between deltoid muscle uptake and age or immune status. Deltoid muscle uptake was associated with only the interval from the second vaccine dose and the number of administered vaccine doses (taking into account that the median time before the PET/CT scan was shorter for the second vaccine dose [4 d] than for the first vaccine dose [11 d]). These associations imply that the high metabolic activity in the lymph nodes might be a marker of vaccine-induced immune system activation, increasing over time and after the second vaccine dose, whereas metabolic activity in the deltoid muscle is inflammatory and likely secondary to local trauma from the injection itself. Whether there is a causal association between axillary lymph node uptake and vaccination-elicited immunogenicity should be further explored.

Our study had a few limitations. First, all patients were vaccinated with the Pfizer-BioNTech COVID-19 vaccine. It is yet unknown whether similar PET/CT findings would be observed after administration of other manufacturers’ mRNA vaccines and whether other vaccine platforms (e.g., viral vectors) would elicit similar uptake on PET/CT. Second, the method used to define patients as immunocompetent versus immunocompromised may have resulted in misclassification of some patients, as we did not account for some conditions requiring immunosuppressive therapy, such as posttransplantation or rheumatologic diseases; however, we assume that their relative proportion in the study cohort was low. Third, as most of the patients underwent ^18^F-FDG PET/CT, the small number of patients undergoing imaging with other tracers (^68^Ga-DOTATATE, PSMA, and ^18^F-DOPA) was not included in the statistical regression model, and more data are required to assess these tracers. Last, the study was conducted during a short period (37 d), and we did not have a repeat scan of the same patients. Although our data show that ^18^F-FDG uptake may be observed long after the first vaccine dose (up to 34 d), we have no data on its trend and change over longer periods. These data could be collected in future studies.

Elderly and high-risk patients are more prone to developing malignancies. Because many countries choose to vaccinate these populations first, and because hundreds of millions of COVID-19 vaccine doses were planned to be administered by the summer of 2021 (*24*), the PET/CT findings we describe here will likely be seen more often. Recognition of reactive axillary lymph node uptake as an indication of prior mRNA vaccination will obviate unnecessary oncologic patient work-up.

## CONCLUSION

In about half the patients receiving the novel mRNA-based COVID-19 vaccine, PET/CT showed avid ipsilateral lymphadenopathy, which was significantly less common in immunocompromised and elderly patients. These findings suggest that ^18^F-FDG PET/CT may hint about the patient’s immune response to the vaccine. In addition, these findings will help inform nuclear medicine physicians about potential PET/CT pitfalls related to COVID-19 vaccination—pitfalls possibly requiring a dedicated intake form addressing recent COVID-19 vaccination. Lastly, these findings may help oncologic physicians in deciding the proper work-up, such as recommending that breast cancer patients be vaccinated on their healthy side to avoid unnecessary biopsies.

## Notes

KEY POINTS **QUESTION:** What is the prevalence of PET/CT findings after COVID-19 mRNA vaccination, and are they associated with the patient’s immune status? **PERTINENT FINDINGS:** In this cross-sectional study of 426 patients after COVID-19 mRNA vaccination, 45% of the 377 who underwent ^18^F-FDG PET/CT showed increased axillary lymph node uptake, which was significantly inversely associated with age and immunosuppressive status. **IMPLICATIONS FOR PATIENT CARE:** Axillary lymph node ^18^F-FDG uptake ipsilateral to the mRNA COVID 19 injection site may hint about the patient’s immune response to the vaccine.

## References

[1] Wang C Horby PW Hayden FG Gao GF. A novel coronavirus outbreak of global health concern. Lancet. 2020;395:470–473. 31986257 10.1016/S0140-6736(20)30185-9 PMC7135038

[2] WHO coronavirus (COVID-19) dashboard. World Health Organization website. https://covid19.who.int. Updated October 5, 2021. Accessed October 6, 2021.

[3] Forni G Mantovani A; COVID-19 Commission of Accademia Nazionale dei Lincei, Rome. COVID-19 vaccines: where we stand and challenges ahead. Cell Death Differ. 2021;28:626–639. 33479399 10.1038/s41418-020-00720-9 PMC7818063

[4] Comirnaty and Pfizer-BioNTech COVID-19 vaccine. U.S. Food and Drug Administration website. https://www.fda.gov/emergency-preparedness-and-response/coronavirus-disease-2019-covid-19/pfizer-biontech-covid-19-vaccine. Updated September 24, 2021. Accessed February 5, 2021.

[5] Moderna COVID-19 vaccine. U.S. Food and Drug Administration website. https://www.fda.gov/emergency-preparedness-and-response/coronavirus-disease-2019-covid-19/moderna-covid-19-vaccine. Updated August 31, 2021. Accessed February 5, 2021.

[6] Interim clinical considerations for use of COVID-19 vaccines currently approved or authorized in the United States. Centers for Disease Control and Prevention website. https://www.cdc.gov/vaccines/covid-19/info-by-product/clinical-considerations.html. Updated September 27, 2021. Accessed February 5, 2021.

[7] Mingos M Howard S Giacalone N Kozono D Jacene H. Systemic immune response to vaccination on FDG-PET/CT. Nucl Med Mol Imaging. 2016;50:358–361. 27994693 10.1007/s13139-015-0385-6 PMC5135690

[8] Panagiotidis E Exarhos D Housianakou I Bournazos A Datseris I. FDG uptake in axillary lymph nodes after vaccination against pandemic (H1N1). Eur Radiol. 2010;20:1251–1253. 20186419 10.1007/s00330-010-1719-5

[9] Thomassen A Lerberg Nielsen A Gerke O Johansen A Petersen H. Duration of ^18^F-FDG avidity in lymph nodes after pandemic H1N1v and seasonal influenza vaccination. Eur J Nucl Med Mol Imaging. 2011;38:894–898. 21340453 10.1007/s00259-011-1729-9

[10] Ayati N Jesudason S Berlangieri SU Scott AM. Generalized lymph node activation after influenza vaccination on ^18^F FDG-PET/CT imaging, an important pitfall in PET interpretation. Asia Ocean J Nucl Med Biol. 2017;5:148–150. 28660226 10.22038/aojnmb.2017.8702 PMC5482920

[11] Burger IA Husmann L Hany TF Schmid DT Schaefer NG. Incidence and intensity of F-18 FDG uptake after vaccination with H1N1 vaccine. Clin Nucl Med. 2011;36:848–853. 21892032 10.1097/RLU.0b013e3182177322

[12] Coates EE Costner PJ Nason MC. Lymph node activation by PET/CT following vaccination with licensed vaccines for human papillomaviruses. Clin Nucl Med. 2017;42:329–334. 28288041 10.1097/RLU.0000000000001603 PMC12366721

[13] Focosi D Caracciolo F Galimberti S Papineschi F Petrini M. False positive PET scanning caused by inactivated influenza virus vaccination during complete remission from anaplastic T-cell lymphoma. Ann Hematol. 2008;87:343–344. 18092164 10.1007/s00277-007-0413-4

[14] Shirone N Shinkai T Yamane T. Axillary lymph node accumulation on FDG-PET/CT after influenza vaccination. Ann Nucl Med. 2012;26:248–252. 22271546 10.1007/s12149-011-0568-x

[15] Williams G Joyce RM Parker JA. False-positive axillary lymph node on FDG-PET/CT scan resulting from immunization. Clin Nucl Med. 2006;31:731–732. 17053400 10.1097/01.rlu.0000242693.69039.70

[16] Patel SY Carbone J Jolles S. The expanding field of secondary antibody deficiency: causes, diagnosis, and management. Front Immunol. 2019;10:33. 30800120 10.3389/fimmu.2019.00033 PMC6376447

[17] Hosmer DW Lemeshow S. Applied Logistic Regression. 2nd ed. John Wiley and Sons; 2000:147–156.

[18] Benjamini Y Hochberg Y. Controlling the false discovery rate: a practical and powerful approach to multiple testing. J R Stat Soc Series B Stat Methodol. 1995;57:289–300.

[19] Chodick G Tene L Patalon T. The effectiveness of the first dose of BNT162b2 vaccine in reducing SARS-CoV-2 infection 13-24 days after immunization: real-world evidence . medRxiv website . https://www.medrxiv.org/content/10.1101/2021.01.27.21250612v1. Published January 29, 2021. Accessed October 6, 2021.

[20] Eifer M Eshet Y. Imaging of COVID-19 vaccination at FDG PET/CT. Radiology 2021;299:E248. 33507123 10.1148/radiol.2020210030 PMC7845454

[21] Castle SC. Clinical relevance of age-related immune dysfunction. Clin Infect Dis. 2000;31:578–585. 10987724 10.1086/313947

[22] Yri OE Torfoss D Hungnes O. Rituximab blocks protective serologic response to influenza A (H1N1) 2009 vaccination in lymphoma patients during or within 6 months after treatment. Blood. 2011;118:6769–6771. 22058114 10.1182/blood-2011-08-372649

[23] Gross PA Gould AL Brown AE. Effect of cancer chemotherapy on the immune response to influenza virus vaccine: review of published studies. Rev Infect Dis. 1985;7:613–618. 3903940 10.1093/clinids/7.5.613

[24] Segers G. Biden says U.S. will have enough vaccine doses for 300 million Americans by end of summer. CBS News website. https://www.cbsnews.com/news/covid-19-vaccine-biden-300-million-doses-summer/. Updated January 26, 2021. Accessed December 7, 2021.

